# Artificial intelligence (AI) models for the ultrasonographic diagnosis of liver tumors and comparison of diagnostic accuracies between AI and human experts

**DOI:** 10.1007/s00535-022-01849-9

**Published:** 2022-02-27

**Authors:** Naoshi Nishida, Makoto Yamakawa, Tsuyoshi Shiina, Yoshito Mekada, Mutsumi Nishida, Naoya Sakamoto, Takashi Nishimura, Hiroko Iijima, Toshiko Hirai, Ken Takahashi, Masaya Sato, Ryosuke Tateishi, Masahiro Ogawa, Hideaki Mori, Masayuki Kitano, Hidenori Toyoda, Chikara Ogawa, Masatoshi Kudo, JSUM A. I. investigators

**Affiliations:** 1 Department of Gastroenterology and Hepatology, Kindai University Faculty of Medicine, 377-2 Ohno-higashi, Osaka-sayama, Osaka, 589-8511 Japan; 2 Department of Human Health Sciences, Graduate School of Medicine, Kyoto University, 53 Kawahara-cho, Shogoin, Sakyo-ku, Kyoto, 606-8507 Japan; 3 Department of Information Engineering, School of Engineering, Cyukyo University, 101 Tokodachi, Kaizu-cho, Toyota, Aichi 470-0393 Japan; 4 Diagnostic Center for Sonography, Hokkaido University Hospital, North 14 West 5, Kita-ku, Sapporo, Hokkaido 060-8648 Japan; 5 Department of Gastroenterology and Hepatology, Faculty of Medicine, Hokkaido University, North 15, West 7, Kita-ku, Sapporo, Hokkaido 060-8638 Japan; 6 Department of Internal Medicine, Division of Gastroenterology and Hepatology, Hyogo College of Medicine, 1-1 Mukogawa-cho, Nishinomiya, Hyogo 663-8501 Japan; 7 Department of General Diagnostic Imaging Center, Nara Medical University Hospital, 840 Shijo-Cho, Kashihara, Nara 634-8522 Japan; 8 Department of Gastroenterology and Hepatology, Graduate School of Medicine, Kyoto University, 53 Kawara-cho, Syogoin, Sakyo-ku, Kyoto, 606-8507 Japan; 9 Department of Clinical Laboratory Medicine, Graduate School of Medicine, The University of Tokyo, 7-3-1 Hongo, Bunkyo-ku, 113-8655 Japan; 10 Department of Gastroenterology, Graduate School of Medicine, The University of Tokyo, 7-3-1 Hongo, Bunkyo-ku, 113-8655 Japan; 11 Department of Gastroenterology, Nihon Univiersity Hospital, 1-6 Kanda-Surugadai, Chiyoda-ku, Tokyo, 101-8309 Japan; 12 Department of Gastroenterology and Hepatology, Kyorin University School of Medicine, 6-20-2 Shinkawa, Mitaka-shi, Tokyo, 181-8611 Japan; 13 Second Department of Internal Medicine, Wakayama Medical University, 811-1 Kimiidera, Wakayama, 641-8509 Japan; 14 Department of Gastroenterology, Ogaki Municipal Hospital, 4-86 Minaminokawa, Ogaki, Gifu 503-8502 Japan; 15 Department of Gastroenterology and Hepatology, Takamatsu Red Cross Hospital, 4-1-3 Ban-cyo, Takamatsu, Kagawa 760-0017 Japan

**Keywords:** Ultrasonography, Liver tumor, Diagnosis, Artificial intelligence, Deep neural network

## Abstract

**Background:**

Ultrasonography (US) is widely used for the diagnosis of liver tumors. However, the accuracy of the diagnosis largely depends on the visual perception of humans. Hence, we aimed to construct artificial intelligence (AI) models for the diagnosis of liver tumors in US.

**Methods:**

We constructed three AI models based on still B-mode images: model-1 using 24,675 images, model-2 using 57,145 images, and model-3 using 70,950 images. A convolutional neural network was used to train the US images. The four-class liver tumor discrimination by AI, namely, cysts, hemangiomas, hepatocellular carcinoma, and metastatic tumors, was examined. The accuracy of the AI diagnosis was evaluated using tenfold cross-validation. The diagnostic performances of the AI models and human experts were also compared using an independent test cohort of video images.

**Results:**

The diagnostic accuracies of model-1, model-2, and model-3 in the four tumor types are 86.8%, 91.0%, and 91.1%, whereas those for malignant tumor are 91.3%, 94.3%, and 94.3%, respectively. In the independent comparison of the AIs and physicians, the percentages of correct diagnoses (accuracies) by the AIs are 80.0%, 81.8%, and 89.1% in model-1, model-2, and model-3, respectively. Meanwhile, the median percentages of correct diagnoses are 67.3% (range 63.6%–69.1%) and 47.3% (45.5%–47.3%) by human experts and non-experts, respectively.

**Conclusion:**

The performance of the AI models surpassed that of human experts in the four-class discrimination and benign and malignant discrimination of liver tumors. Thus, the AI models can help prevent human errors in US diagnosis.

**Supplementary Information:**

The online version contains supplementary material available at 10.1007/s00535-022-01849-9.

## Introduction

With the increase of the aged population, the prevalence of malignant liver tumors is expected to increase, so an efficient method for screening lesions is imperative [1, 2]. Abdominal ultrasonography (US) is a non-invasive, highly convenient, and versatile imaging technique that is commonly used for liver tumor diagnosis. However, extensive experience in US is required for accurate diagnoses because of the need to perform real-time recognition of lesions [3]. From this viewpoint, the lack of experts in US is an urgent issue in the medical sector that needs to be addressed because missing and incorrect diagnoses by non-experts will have serious consequences.

Artificial intelligence (AI) is emerging as a major tool in the fields of medicine and healthcare, particularly in image diagnosis [4, 5]. It is easily applied to imaging data because AI excels in recognizing unique and complex image features and facilitates quantitative assessments [6]. This unique characteristic of AI is ideal for constrained clinical settings where a medical professional needs to evaluate a large number of images using visual perception, with some uncertainties and inevitable human errors. As a result, AI has been applied to many aspects of medical imaging, such as computed tomography (CT), magnetic resonance imaging (MRI), colonoscopy, mammography, and pathological assessment [5–10].

Several reports have been published on machine learning to assess the diagnosis of liver tumors. Recently, deep neural networks have become available in the field of imaging diagnostics. In this context, US supported by AI will be an ideal device for screening liver lesions, particularly for the early diagnosis of liver cancer [11]. However, several issues still need to be resolved before the application of AI in US diagnosis because of the specific nature of US image. For instance, US images are heterogeneous with a variety of features, even for the same type of lesions. US images can be affected by the location of the lesion, such as the depth from the body surface, and the patient’s position. In addition, the difference in image processing, which could vary among equipment, gives rise to heterogeneity in images. Therefore, a number of heterogenous image datasets from several institutes are required to train a neural network to produce a reliable AI model that can be applied to a clinical setting, which has not yet been established [12].

In this study, AI models were built using a large number of B-mode images of liver tumors. We compared the performance of three AI models pretrained with 24,675 images, 57,147 images, and 70,950 still B-mode images to know the improvement of diagnostic accuracies in liver tumor discrimination based on the increase of training data. We also compared the diagnostic accuracies between the AI models and physicians using independent external test cohorts of video images of liver tumors. We confirmed that the accuracy of AI in discriminating liver tumors exceeds that of human experts, indicating that AI models have sufficient potential for applications in clinical settings.

## Materials and methods

### Dataset for the training, validation, and test cohorts of the AI models

This is a multicenter diagnostic study using the B-mode ultrasound image dataset of liver tumors. The inclusion criteria for data collection are as follows: (1) Patients’ liver tumors were detected by B-mode US. (2) The diagnosis of the liver tumor was confirmed via histology, contrast-enhanced computed tomography (CECT), contrast-enhanced ultrasonography using Sonzoid® (CEUS), or gadolinium-ethoxybenzyl-diethylenetriamine pentaacetic acid (Gd-EOB-DTPA)-enhanced MRI.

In this study, 94,427 B-mode images of liver tumors from 29,264 cases were collected from April 1, 2018 to March 12, 2021 in 11 core medical centers across Japan. Of these images, 25,495 still B-mode images from 8712 cases of liver tumors were available by January 20, 2020, where 24,675 images of four types of liver tumors, namely, hepatocellular carcinoma (HCC), metastatic liver tumor (metastatic tumor), liver hemangioma (hemangioma), and liver cyst (cyst), from 8585 cases were used to construct AI model-1 for the liver tumor discrimination. Similarly, 59,196 still B-mode images from 20,424 cases of liver tumors were available by June 29, 2020, of which 57,145 images of the four types of liver tumors from 20,318 cases were used for AI model-2. Then, 80,258 images from 25,779 cases were available by December 17, 2020, of which 70,950 still B-mode images from 23,756 cases were used for AI model-3. The remaining 821 images from 127 cases, 2,015 images from 106 cases, and 9,308 images from 2023 cases for model-1, model-2, and model-3 were excluded from the analysis because they present other types of tumors (Supplementary Fig. 1). We also applied 4,633 images from 972 cases for developing an AI model that help classify another set of tumors: HCC, intrahepatic cholangiocarcinoma (ICC), metastatic tumor, and hemangioma (model-ICC). More details on the dataset are described in Supplementary Materials and Methods.

There were no restrictions on the manufacturer or model of equipment for the collection of the B-mode images. All the images were collected based on the system construction guidelines for the collection of medical images published by the Japan Association for Medical Informatics (http://jami.jp/about/documents/amed_report.pdf). The study was conducted in accordance with the World Medical Association Declaration of Helsinki. The study protocol conformed to the ethical guidelines of the 1975 Declaration of Helsinki. Informed consent was waived for retrospectively collected data in the medical records, including US images, if they were anonymized. The study was approved by the ethics committees of all participating institutions.

### Image processing

We cropped a region of interest (ROI) that included a liver tumor from the entire ultrasound image as a preprocessing step for training a convolutional neural network (CNN). A square ROI was used because a square image is generally applied as an input image to the CNN and the aspect ratio of the tumor does not change in the input image. Furthermore, in ultrasonic diagnosis, aside from information inside the tumor, information around the tumor is also critical for diagnosis. In a previous study, we examined the size of the surrounding area that should be included in the ROI for the most accurate liver tumor classification [13]. We reported that the best accuracy was achieved when the ROI was cropped so that the maximum diameter of the tumor was 0.6 times the ROI size, as shown in Supplementary Fig. 2. Accordingly, the same criterion was used in this study.

In the database used for training the AI model-1, model-2, and model-3 the amount of data for HCC, metastatic tumors, hemangiomas, and cysts was different, and the number of datasets for HCC and metastatic tumors was smaller than that for hemangiomas and cysts. Therefore, to avoid a biased output that is prone to being classified as a hemangioma or cyst, we performed data augmentation so that the number of training data for each liver tumor type is almost equal. For the data augmentation, we added left–right reversed images and rotated images with limited angles to the training data. In general, the rotation of an ultrasound image is not desirable because it is direction dependent. However, because a convex probe is used in abdominal ultrasonography, the ultrasonic beam is tilted within a range of approximately ± 30°. Therefore, we added randomly rotated images in the range of ± 10° to the training data. Using the augmented data, we upsampled the HCC and metastatic tumor data by a factor of four and the hemangioma and cyst data by a factor of two. Finally, the images were resized to the input image size of the CNN.

On the other hand, for the development of AI model-ICC, the number of the original images of ICC was too small compared to those of HCC, metastatic tumor and hemangioma. Therefore, to avoid a bias based on the disproportion in the amount of original data among tumor types, we downsized the number of the images for HCC, metastatic tumor, and hemangioma to the similar amount of ICC images for training.

### Construction of AI models using CNN

**Fig. 1:**
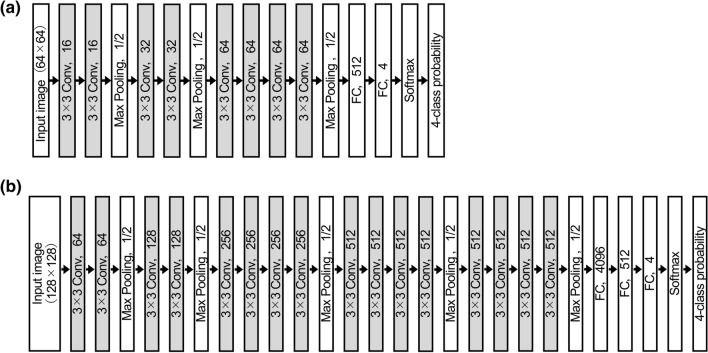
Convolutional neural network (CNN) used in the AI models. **a** CNN used in AI model-1 and AI model-ICC. This CNN is based on VGGNet. It was used when the number of datasets was small. The input image size of the CNN is 64 × 64 pixels, and the output is the probability of the four classes (HCC, metastatic tumors, hemangiomas, and cysts). **b** CNN used in AI model-2 and model-3. This CNN was used when the number of datasets was large. The CNN is also based on VGGNet. The input image size of the CNN is 128 × 128 pixels, and the output is the probability of the four classes (HCC, metastatic tumors, hemangiomas, and cysts). The “3 × 3 Conv, 16” represents a convolutional layer with a filter size of 3 × 3 and 16 channels. Although omitted in the figure, a batch normalization layer and rectified linear unit function were also included after all the convolutional layers. “Max Pooling, 1/2” represents a max pooling layer that halves the image size, and “FC, 4” represents a fully connected layer having four outputs

CNNs based on VGGNet were used in the AI models as shown in Fig. 1. Figure 1a shows the CNN applied for model-1 and model-ICC, and Fig. 1b shows the CNN for large-scale training datasets used for model-2 and model-3 to improve the accuracy. We used adaptive moment estimation (Adam) as the solver during training and set the learning rate to 0.001. The optimum number of epochs was found to be between 10 and 30 using the validation data. The *k*-fold cross-validation method (*k* = 10) was used to validate the CNN models. That is, all the datasets were divided into 10 groups, one of which was used as test data, another as validation data, and the remaining eight as training data. Then, the groups used for testing, validation, and training were changed so that all groups would be used once as test data.

## Comparative study of the discrimination of liver tumors between the AI models and physicians

### Dataset for a comparative study

A comparative study was conducted using video images of 55 patients with liver tumors. The test dataset used for the comparison of AI and human physicians was completely different from that used for cross-validation. All tumors used in this study were of the nodular type with a visible boundary. Video images showing the entire picture of a tumor were selected for comparison between AI models and humans.

### Diagnosis of tumors by AI and physicians

The diagnosis of four types of tumors was performed by physicians using video images. Eight physicians participated in this study: five were experts who were qualified as specialists by the academic societies (expert group), whereas the remaining three were non-specialists (non-expert group). There was no time restriction for the physicians’ answers.

For the AI diagnosis, five different still frames showing tumors were used for each nodule: one was a frame showing the tumor with the maximum diameter where the median size of the tumors is 22 mm in the major axis (range 6–75), two showing tumors that are 75% of the maximum diameter, and two with tumors that are 50% of the maximum diameter. The sites of the tumors were selected as the ROIs in the frame and applied for AI diagnosis, as described above. Among the four types of tumors, the diagnosis with the highest estimated probability in three or more frames was selected as the AI diagnosis (≥ 3 out of the five-frame rule). More details on the dataset for the comparison test, AI models applied, physicians involved in the comparison test, and judgment rule for the correct diagnosis by AI are presented in Supplementary Materials and Methods.

## Statistics

We compared the diagnostic accuracy, which was calculated as the number of nodules with correct estimation divided by the number of the total test nodules among AI model-1, model-2, model-3, and physicians. Sensitivity and specificity were also compared for the two-class classification of nodules. For the comparison of performance among the three AI models, we applied nonparametric multiple comparisons using the Steel–Dwass test. For the comparison of the AIs and human experts, we used the nonparametric Wilcoxon rank sum test.

## Results

### Accuracy of the diagnosis of liver tumors in AI model-1, model-2, and model-3

As described above, three CNN AI models, model-1 with 24,675 images, model-2 with 57,147 images, and model-3 with 70,960 images of liver tumors, were constructed. The accuracies of the AI models in the diagnosis of the four types of tumors (4-class discrimination) were 86.8%, 91.0%, and 91.1% for model-1, model-2, and model-3, respectively; the overall accuracy for four-class discrimination improved steadly with the increase of training data, indicating the CNN algorism was effectively trained with US imagess (Supplementary Fig. 3). The respective diagnostic accuracies of model-1, model-2, and model-3 in each type of tumor were as follows: 89.9%, 93.2%, and 93.4% for the diagnosis of HCC; 93.4%, 95.2%, and 95.1% for the diagnosis of metastatic tumors; 91.2%, 94.6%, and 94.6% for the diagnosis of hemangiomas; and 98.5%, 98.9%, and 99.0% for the diagnosis of cysts (Table 1, Supplementary File 1). The sensitivities for the diagnosis in each type of tumor were 64.6%, 68.1%, and 67.5% for HCCs; 62.0%, 60.6%, and 62.8% for metastatic tumors; 87.5%, 90.8%, and 91.0% for hemangiomas; and 98.3%, 99.1%, and 99.0% for the diagnosis of cysts by model-1, model-2, and model-3, respectively. Similarly, the specificities for each tumor are 93.8%, 96.0%, and 96.0% for HCCs; 96.7%, 97.4%, and 97.5% for metastatic tumors; 93.9%, 96.6%, and 96.5% for hemangiomas; and 98.7%, 98.8%, and 98.8% for cysts by model-1, model-2, and model-3, respectively. Compared to AI model-1, all accuracies, all sensitivities but for metastatic tumors, and all specificities are higher in model-2. Similally, all accuracies, sensitivities and specificities for the diagnosis of these four types of tumors are higher in model-3 compared to model-1 (Table 1, Supplementary File 1). The accuracies of model-1, model-2, and model-3 for the diagnosis of malignant tumors were 91.3%, 94.3%, and 94.3%, respectively, whereas the sensitivities and specificities were 80.9% and 94.4% by model-1, 82.4% and 96.8% by model-2, and 82.8% and 96.7% by model-3, respectively (Table 2).

**Table 1:** The performance for each diagnosis for liver tumor by AI modes

True diagnosis	HCC			Metastatic tumor			Hemangioma			Cyst		
AI diagnosis	Model-1	Model-2	Model-3	Model-1	Model-2	Model-3	Model-1	Model-2	Model-3	Model-1	Model-2	Model-3
HCC	**833**	**1532**	**1750**	160	300	326	231	325	362	20	33	48
Metastatic tumor	181	295	369	**430**	**767**	**938**	98	137	201	22	42	34
Hemangioma	250	379	433	91	173	204	**2465**	**4967**	**6109**	22	31	45
Cyst	25	43	41	12	25	25	24	44	38	**3721**	**11,225**	**12,833**
Accuracy^a^	**89.9%**	**93.2%**	**93.4%**	**93.4%**	**95.2%**	**95.1%**	**91.2%**	**94.6%**	**94.6%**	**98.5%**	**98.9%**	**99.0%**
Sensitivity^b^	**64.6%**	**68.1%**	**67.5%**	**62.0%**	**60.6%**	**62.8%**	**87.5%**	**90.8%**	**91.0%**	**98. 3%**	**99.1%**	**99.0%**
Specificity^c^	**93.8%**	**96.0%**	**96.0%**	**96.7%**	**97.4%**	**97.5%**	**93.9%**	**96.6%**	**96.5%**	**98.7%**	**98.8%**	**98.8%**

The numbers of the nodule for true and estimated diagnosis by model-1, model-2, and model-3 are shown for HCC metastatic liver tumor, liver hemangioma, and liver cyst. Bold indicates the number of the tumor that shows a correct answer by AI

Details for the calculations of the sensitivity and specificity for the diagnosis of each type of tumor are shown in supplementary file 1

^a^Accuracy for diagnosis is calculated as number of the tumors with correct estimations divided by the number of total tumors

^b^Sensitivity for diagnosis of HCC is calculated as the number of the tumor estimated as HCC by AI divided by the number of the true HCC. Sensitivities for other types of tumors are calculated similarly

^c^Specificity for diagnosis of HCC is calculated as the number of the nodule estimated as non-HCC by AI divided by the number of the true non-HCC nodules. Specificity for other types of tumors are calculated similarly

**Table 2:** The sensitivity and specificity for diagnosing malignant tumor by AI models

True diagnosis	Benign				Malignant		
AI diagnosis	Model-1	Model-2	Model-3		Model-1	Model-2	Model-3
Benign	**6232**	**16,267**	**19,025**		378	620	703
Malignant	371	537	645		**1604**	**2894**	**3383**
Specificity^a^	94.4%	96.8%	96.7%	Sensitivity^a^	80.9%	82.4%	82.8%

The numbers of the nodule for true and estimated diagnosis by model-1, model-2, and model-3 are shown for benign (hemangioma and cyst) and malignant tumors (HCC and metastatic liver tumor). Bold indicates the number of the tumor that shows a correct answer by AI

^a^Specificity and specificity for diagnosing malignant tumor. The accuracy of diagnosis is 91.3%, 94.3%, and 94.3%, respectively by model-1, model-2, and model-3 which is calculated as total number of estimated correct answers by AI divided by the number of total cases

We also evaluated the diagnostic performace of AI model-ICC using the *k*-fold cross-validation (*k* = 10). The overall accuracy for four-class discrimination is 71.5%, where accuracies for diagnosing HCC, ICC, metastatic tumor and hemangioma are 83.3%, 88.6%, 83.0%, and 88.1%, respectively. The accuracies, sensitivities, and specificities for diagnosing each type of tumors are also shown in Supplementary File 2. Similarly, the accuracy, sensitivity, and specificity for diagnosing malignant tumor are 88.1%, 90.5%, and 83.1%, respectively.

## Comparison of the diagnostic accuracies of AI and physicians

We compared the performances of the three AI models (model-1, model-2, and model-3) and eight physicians, which included five experts certified by academic societies and three non-experts, for the diagnosis of liver tumor using 55 videos of tumors (i.e., HCC, metastatic tumors, hemangiomas, and cysts). The list of diagnoses for the 55 test cases in the comparative study and the probabilities for the correct diagnosis estimated by the AI models in five frames and diagnoses by physicians are shown in Supplementary File 3.

**Fig. 2:**
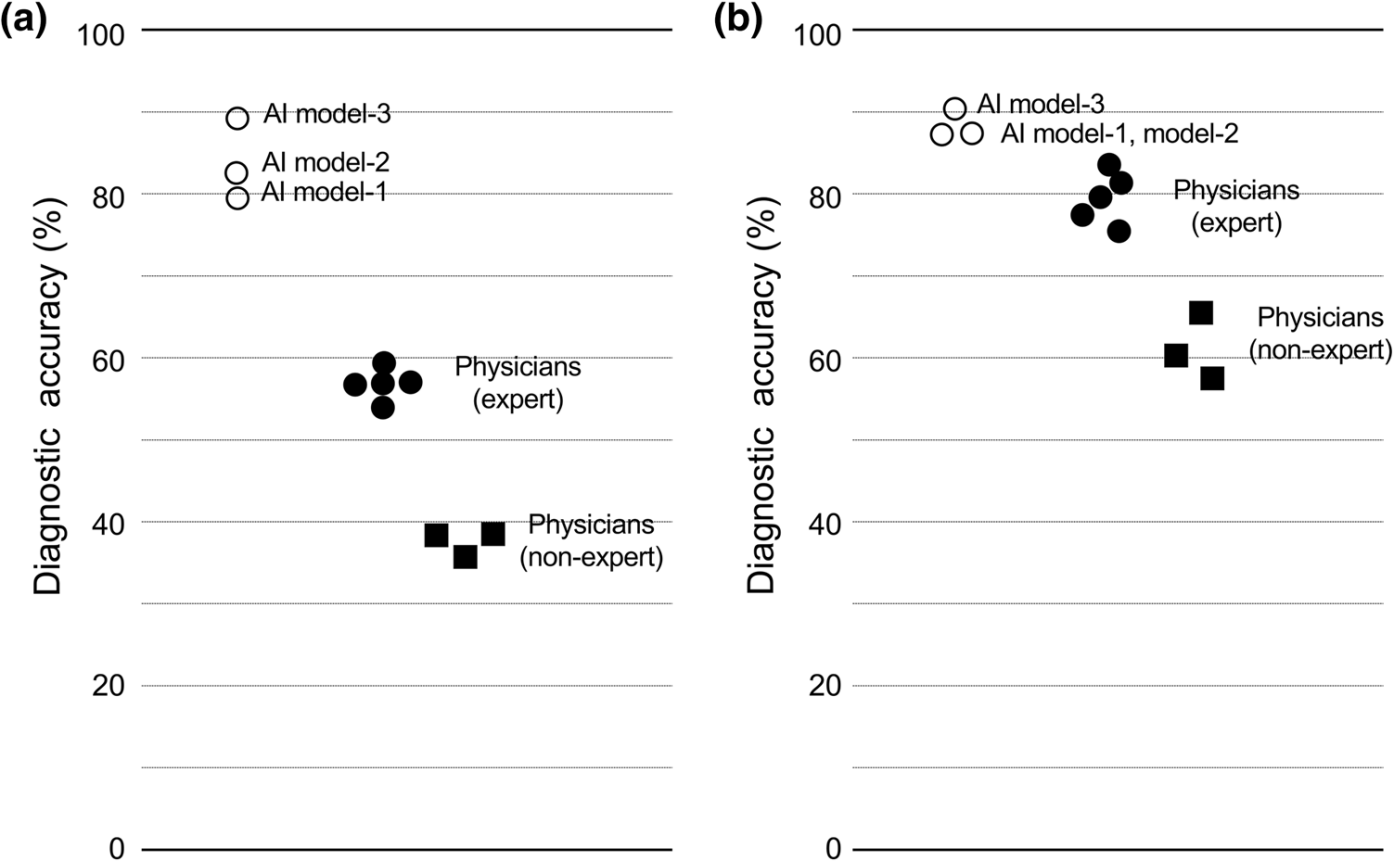
Comparison of the diagnostic accuracies between AI and human physicians. Diagnostic accuracies for the four-class discrimination and benign and malignant tumor discrimination were compared among the three AI models, five human experts, and three non-experts. **a** Comparison of diagnostic accuracies for the four-class discrimination. **b** Comparison of diagnostic accuracies for the malignant tumor. Open circle indicates the accuracies of AI model, solid circle shows accuracies of the human experts, and solid square shows accuracies of the non-experts. The details of each value are shown in Supplementary File 3

The overall diagnostic accuracies of model-1, model-2, and model-3 were 80.0% (44/55), 81.8% (45/55), and 89.1% (50/55), respectively. The median percentages of the diagnostic accuracies of physicians were 67.3% (range 63.6%–69.1%) and 47.3% (45.5%–47.3%) for experts and non-experts, respectively (Fig. 2a, Supplementary File 4). Because each AI model yielded only one value of accuracy (percentage of correct answer), we compared accuracies of three AI models with those of 5 human experts. The accuracies of AI models are significantly higher than those of human experts (*p* = 0.0325 by Wilcoxon rank sum test). The accuracies in diagnosis of HCC, metastatic tumors, hemangiomas, and cysts by AI models were as follows: 85.5%, 87.3%, 90.9%, and 96.4% in model-1; 87.3%, 89.1%, 90.9%, and 96.4% in model-2; and 92.7%, 94.6%, 98.2%, and 96.4% in model-3, respectively (Table 3). For the eight human physicians, the median diagnostic accuracies in each type of tumor are as follows: 69.1% (range 74.5 – 61.8), 80.9% (85.5 – 63.6), 80.0% (83.6 – 56.4), and 97.3% (100 – 94.6) for HCC, metastatic tumors, hemangiomas, and cysts, respectively (Supplementary File 4). The sensitivities and specificities for the diagnosis of each type of tumor by the AI models and human physicians are also detailed in Supplementary File 4. All the accuracies, sensitivities and specificities for the diagnosis of these types of liver tumors, except for cyst, by the AI models are higher than the corresponding values by the human physicians including experts. The sample US images that showed the differential results between AI model-3 and human experts are shown in Supplementary Fig. 4.

**Table 3:** The performance of diagnosis for each type of tumor by AI model-1, model-2, and model-3 in the comparative test

True diagnosis	HCC			Metastatic tumor			Hemangioma			Cyst		
AI diagnosis	Model-1	Model-2	Model-3	Model-1	Model-2	Model-3	Model-1	Model-2	Model-3	Model-1	Model-2	Model-3
HCC	**11**	**13**	**14**	1	2	0	0	0	0	0	0	0
Metastatic tumor	3	1	1	**5**	**4**	**7**	0	0	0	0	0	0
Hemangioma	4	4	1	1	1	0	**23**	**23**	**23**	0	0	0
Cyst	0	0	0	2	2	2	0	0	0	**5**	**5**	**5**
Not determined^a^	0	0	2	0	0	0	0	0	0	0	0	0
Accuracies^b^	**85.5%**	**87.3%**	**92.7%**	**87.3%**	**89.1%**	**94.6%**	**90.9%**	**90.9%**	**98.2%**	**96.4%**	**96.4%**	**96.4%**
Sensitivity^c^	**61.1%**	**72.2%**	**77.8%**	**55.5%**	**44.4%**	**77.8%**	**100%**	**100%**	**100%**	**100%**	**100%**	**100%**
Specificity^d^	**83.7%**	**87.5%**	**90.2%**	**91.4%**	**90.0%**	**95.7%**	**100%**	**100%**	**100%**	**100%**	**100%**	**100%**

The numbers of the nodule for true and estimated diagnosis by model-1, model-2, and model-3 are shown for HCC metastatic liver tumor, liver hemangioma, and liver cyst. Bold indicates the number of the tumor that shows a correct answer by AI

Details for the calculations of the sensitivity and specificity for the diagnosis of each type of tumor are shown in supplementary file 3

^a^No specific diagnoses are not made by AI with " ≥ 3 out of the 5-frame rule", because no consistent diagnosis with the highest estimated probability is shown in three or more frames among 5 frames

^b^Accuracy for diagnosis is calculated as number of the tumors with correct estimations divided by the number of total tumors

^c^Sensitivity for diagnosis of HCC is calculated as the number of the nodule estimated as HCC by AI divided by the number of the true HCC. Sensitivities for other types of tumors are calculated similarly

^d^Specificity for diagnosis of HCC is calculated as the number of the nodule estimated as non-HCC by AI divided by the number of the true non-HCC nodules. Specificities for other types of tumors are calculated similarly

Conversely, the diagnostic accuracies for malignant tumors by the AIs were 87.3% (48/55) for model-1, 87.3% (48/55) for model-2, and 90.9% (50/55) for model-3 (Fig. 2b, Supplementary File 4). For the human physicians, the median the diagnostic accuracy among the test nodules was 80.0% (range 74.5%–83.6%) by experts and 60.0% (range 52.7%–65.5%) by non-experts. The accuracies of AI models are also significantly higher than those of human experts (*p* = 0.0358 by Wilcoxon rank sum test). The sensitivities and specificities for the diagnosis of malignant tumors were 74.1% (20/27) and 100% (28/28) for model-1, 74.1% (20/27) and 100% (28/28) for model-2, and 81.5% (22/27) and 100% (28/28) for model-3, respectively. For the human physicians, the median sensitivity and specificity were 77.8% (66.7%–85.2%) and 82.1% (75.0%–92.9%) by the experts and 70.4% (66.7%–88.9%) and 42.9% (35.7%–53.6%) by the non-experts, respectively (Supplementary File 4).

## Probabilities of correct diagnoses by the AI models

**Fig. 3:**
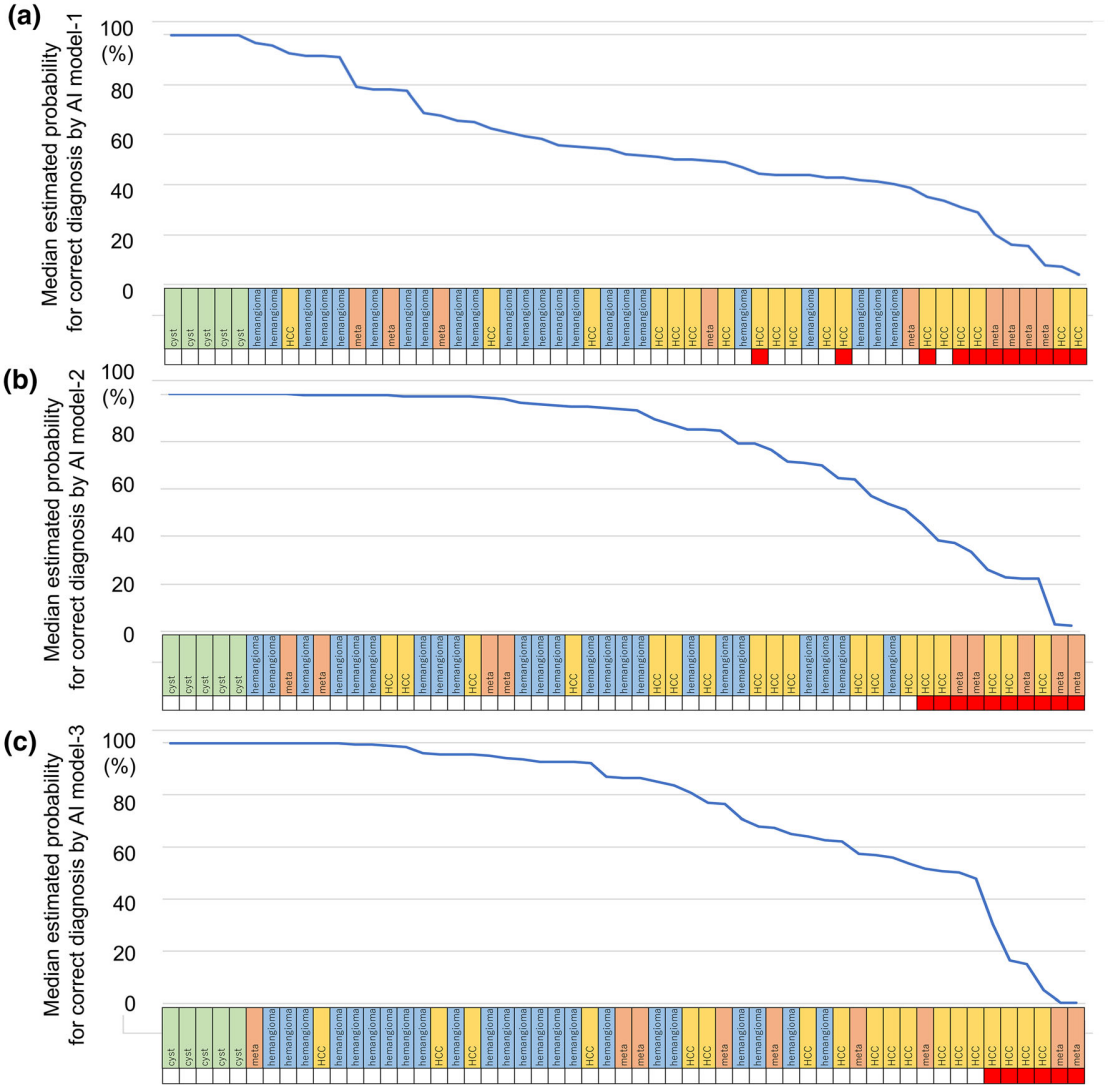
Difference in the median estimated probabilities for the correct diagnosis by AI model-1, model-2, and model-3 in each nodule. The median estimated probabilities for the correct diagnosis for the five frames are shown in a descending order for each nodule; the blue line shows the median probabilities estimated by AI for each nodule. **a** The median estimated probabilities for correct diagnoses by AI model-1. **b** Median estimated probabilities for correct diagnoses by AI model-2. **c** Median estimated probabilities for correct diagnoses by AI model-3. The green, blue, yellow, and pink rectangles represent the tumor types, namely, cysts, hemangiomas, HCCs, and metastatic tumors, respectively. The red rectangles represent the nodules incorrectly estimated for diagnosis by AIs based on the “ ≥ 3 out of the 5-frame rule,” where 80.0% (44/55) of the nodules, 81.8% (45/55) of the nodules, and 89.1% (49/55) of the nodules were correctly diagnosed by AI model-1, model-2, and model-3, respectively

**Fig. 4:**
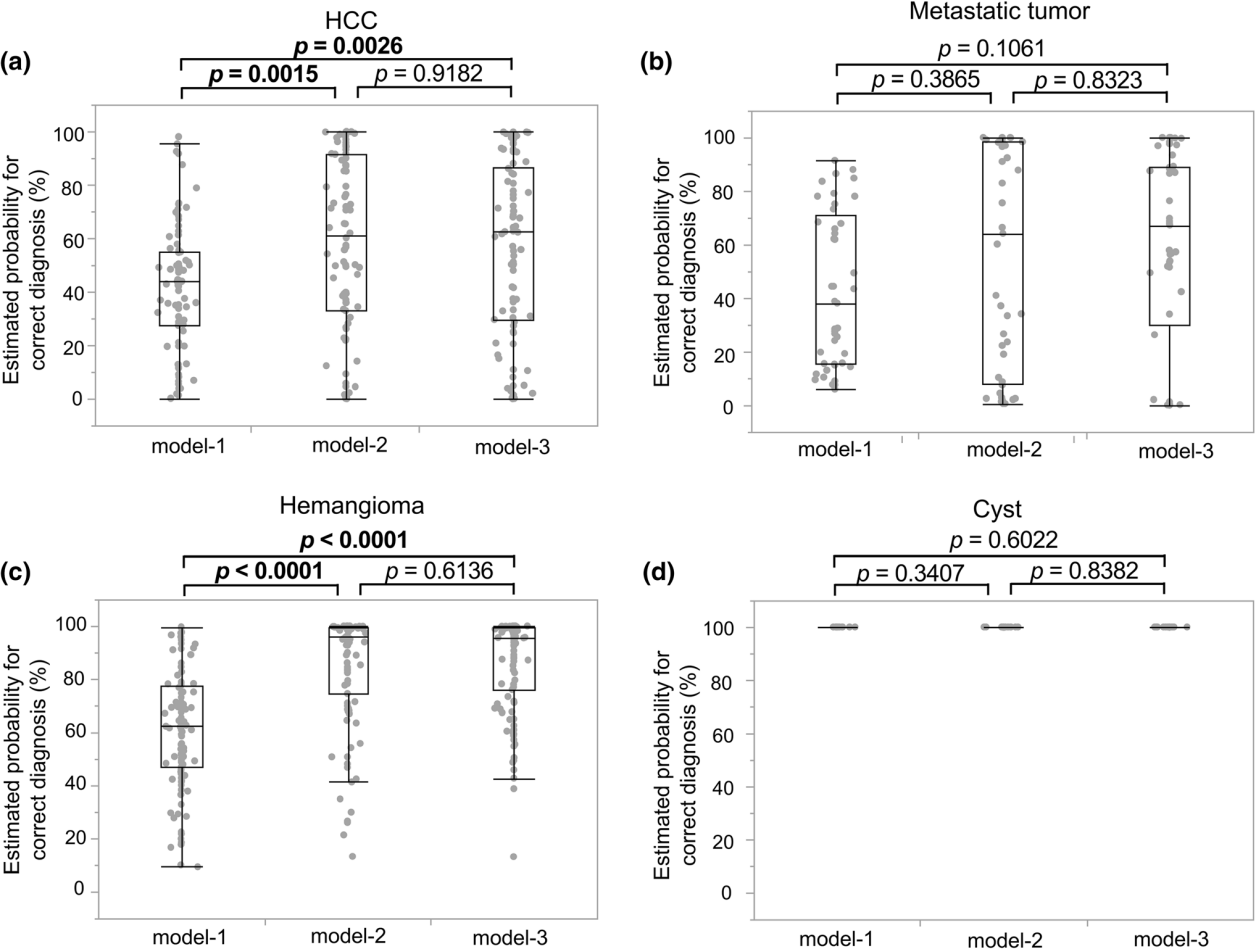
Comparisons of the estimated probabilities for correct diagnoses by the three AI models. The estimated diagnostic probabilities of each tumor type were compared using the estimations in each frame of 55 test nodules. **a** Estimated probability for HCC; **b** estimated probability for metastatic tumors; **c** estimated probability for hemangiomas; **d** estimated probability for cysts. The probabilities of the correct diagnoses of HCC and hemangiomas significantly improved in model-2 and in model-3 as compared with model-1, where the median estimated probability is 43.9% (25–75 percentiles = 27.6–54.8), 61.5% (25–75 percentiles = 32.9–91.6), and 62.3% (25–75 percentiles = 29.3–86.6) for the diagnosis of HCC by model-1, model-2, and model-3, respectively (*p* = 0.0015 for model-1 vs. model-2 and *p* = 0.0026 for model-1 vs. model-3 in the nonparametric multiple comparisons and Steel–Dwass test). Similarly, the median estimated probability was 62.7% (25–75 percentiles = 47.2–77.6), 96.2% (25–75 percentiles = 74.6–99.7), and 95.3% (25–75 percentiles = 76–99.4) for the diagnosis of hemangiomas by model-1, model-2, and model-3, respectively (*p* < 0.0001 for model-1 vs. model-2, and *p* < 0.0001 for model-1 vs. model-3 by the Steel–Dwass test). Although the estimated probabilities by model-2 and model-3 are higher than that of model-1 for the diagnosis of metastatic tumors, the difference is not significant (*p* = 0.3865 for model-1 vs. model-2 and *p* = 0.1061 for model-1 vs. model-3: median estimated probabilities [25–75 percentiles] were 38.1% [15.4–70.8], 64.1% [8.1–98.6], and 67.2% [30.2–89.0] by model-1, model-2, and model-3, respectively). The median probabilities for the diagnosis of cysts were 100% in all estimations

We hypothesized that the estimation for a correct diagnosis could be further improved in AI model-2 and model-3 as compared to model-1, where a larger number of US images were applied for the training of the CNN model. Accordingly, we compared the third-highest estimated probability for a correct diagnosis (the median value of the estimated probabilities for a correct answer among five frames). Figure 3 illustrates the difference in the median estimated probabilities for the correct diagnosis of model-1, model-2, and model-3 in each nodule, where 90.9% (50/55) and 89.1% (49/55) of the nodules showed a higher estimated probability in model-2 and model-3 than in model-1, respectively. This finding suggests that estimating the correct diagnosis based on the probability is improved according to the increase in the training data. The estimated diagnostic probabilities of each tumor type were also compared using the estimations of each 5 frame in 55 test nodules (Fig. 4). The estimation for hemangiomas significantly improved in model-2 and model-3 as compared with model-1 (*p* < 0.0001 for model-1 vs. model-2 and *p* < 0.0001 for model-1 vs. model-3 by the Steel–Dwass test: median estimated probabilities and [25–75 percentiles] were 62.7%, [47.2–77.6] for model-1, 96.2%, [74.6–99.7] for model-2, and 95.3%, [76–99.4] for model-3, respectively). Similarly, the diagnostic probability for HCC was significantly improved in model-2 and model-3 as compared with model-1 (*p* = 0.0015 for model-1 vs. model-2 and *p* = 0.0026 for model-1 vs. model-3: median estimated probabilities [25–75 percentiles] were 43.9%, [27.6–54.8] for model-1, 61.5%, [32.9–91.6] for model-2, and 62.3%, [29.3–86.6] for model-3). The probability of diagnoses for cysts was the same for all the models, where the median probability (25–75 percentiles) was 100% in all the estimations. For metastatic tumors, although the estimated probability improved after the increase of training, the differences between model-1 and model-2 and between model-1 and model-3 were not significant (*p* = 0.3865 for model-1 vs. model-2 and *p* = 0.1061 for model-1 vs. model-3: median estimated probabilities [25–75 percentiles] are 38.1% [15.4–70.8] for model-1, 64.1% [8.1–98.6] for model-2, and 67.2% [30.2–89.0] for model-3).

## Discussion

Liver cancer is one of the leading causes of cancer deaths, and screening for early detection of lesions is recommended for high-risk groups in clinical guidelines [14, 15]. US is commonly used for early detection of liver tumors. However, although it is the most popular imaging equipment, the diagnosis of lesions using B-mode ultrasound is sometimes difficult, particularly for beginners and non-experts, as it requires real-time judgment by a human’s visual perception, and omissions and misdiagnoses due to human errors can occur, especially under the time constraint of clinical workflows [3, 16, 17]. Accordingly, the use of AI can reduce errors because of its high performance in large-volume screening of medical images and the precise detection of subtle image features [12].

Several AI systems for medical images have been proposed, such as detection of referable diabetic retinopathy on retinal fundus images, lymph node metastasis of breast cancer on microscopic slide images, skin cancer on demography, pneumonia on radiography, and neoplastic polyps on colonoscopy [7, 10, 18, 19].

For abdominal US, most reported AI models were for the diagnosis of liver tumors, although the targeted lesions are varied among studies [11, 12, 20]. So far, AI models for liver tumor diagnosis have been based on small cohorts of data (Supplementary File 5). Hwang et al. showed that AI model pretrained with 99 US images of liver tumor achieved 96% of overall accuracy for the discrimination of cyst, hemangioma, and malignant tumor [21]. Tarek et al., reported the AI model pretrained with 108 images showed 97.2% of overall accuracy for discrimination of normal liver, cyst, hemangioma, and HCC [22]. Virmani et al., reported an AI model that showed 95% of accuracy for discrimination of lesions including cyst, hemangioma, HCC, and metastatic tumor [23]. However, although the accuracies are high even with small number of training data, these evaluations are based on the cross-validation; a similar performance might not be expected in an independent cohort, especially for multiclass classification, because of the lack of variation in the training images [11, 12]. On the other hand, Zhang et al., reported the AI model developed using transfer learning form the CEUS image and achieved the diagnostic accuracy of 88.2% [24]. In our study, the overall accuracy of model-2 and model-3 in the four-class discrimination were 91.0% and 91.1%, respectively, which are comparable or even higher than those reported in previous studies. Compared with the previous study, much larger size of the training cohort with enough variation from 11 different institutes should give rise to the reliable performance of our AI models even in the independent test cohort, which should not be affected by overfitting [5].

The accuracy of the discrimination for the four types of tumors based on the tenfold cross-validation method was better in model-2 and model-3 than in model-1. For the diagnosis of malignant tumor, the accuracy, sensitivity, and specificity were also higher in model-2 and model-3 than in model-1. For the diagnosis of each tumor, model-2 and model-3 showed higher accuracies, sensitivities and specificities in the discrimination of each type of liver tumor than model-1. However, the sensitivity levels remained similar in metastatic tumors. The lack of improvement for the sensitivity of metastatic tumors is probably attributed to the variation in this type of tumor, which is generally dependent on the origin of the disease; increasing the training volume would improve the diagnostic performance in this type of tumors.

Diagnosis of malignant tumors is quite important for the selection of patients who require further examinations [14–16], where a final diagnosis is generally made by other modalities, such as CECT, Gd-EOB-DTPA-enhanced MRI, and histology [14, 15]. According to three previous reports of CEUS-based AI, the accuracies for diagnosing malignant tumors were 90.3%, 91.8%, and 90.4% [25–28]. Although our AI models were trained using B-mode images, the diagnostic accuracies for malignant tumors were 91.3%, 94.3%, and 94.3% in model-1, model-2, and model-3, respectively, which are higher than the previous AIs trained with CEUS images. The high performance of our B-mode AI models is attributed to the unprecedented volume of data for the training of deep CNNs.

Recently, another study reported that an AI model trained with 24,343 B-mode US images of liver tumors from 2,143 cases showed a similar diagnostic accuracy for malignant tumor with experienced radiologists. The AI model trained with US images and clinical data, including serum levels of tumor markers, reportedly, represented better accuracy, sensitivity, and specificity for the diagnosis of malignant tumor than a skilled radiologist [20]. The AI models trained with B-mode image showed a diagnostic accuracy of 76.0%, and those trained with B-mode image and clinical data represented an accuracy of 84.7% [20]. Our AI model-3 showed a higher diagnostic accuracy for malignant tumor: 94.3% in the tenfold cross-validation and 90.1% in the independent external validation cohort (Supplementary File 4). Therefore, our high-performance AI models should be quite applicable in a clinical setting without additional clinical data, which would fluctuate at the timing of sampling.

We also constructed the AI model for diagnosis of ICC (model-ICC), which has not been reported. ICC is a rare type of cancer that accounts for approximately only 3—5% of primary liver cancer; there is a lack of training images for ICC compared to those of HCC, metastatic tumor, and hemangioma. To avoid a biased output that prone to classify lesions into other common type of tumors, we downsized the numbers of images from HCC, metastatic tumor and hemangioma, and matched these numbers to that of ICC for training. Although the performance of AI model-ICC for four-class discrimination is still unsatisfactory, where the overall accuracy is 71.5%, and diagnostic accuracies for HCC, ICC, metastatic tumor and hemangioma are 83.3%, 88.6%, 83.0%, and 88.1%, respectively, indicating the potential of model-ICC with further training. In addition, the accuracy, sensitivity, and specificity for diagnosis of malignant tumor are 88.1%, 90.5%, and 83.1%, respectively. Because benign and malignant discrimination is critical for the management of liver tumor, and difficult for beginners, discrimination of ICCs by AI model is also an attractive task.

To confirm the robustness of the performance of our AI models, we conducted a comparative study for the diagnosis between AIs and human physicians using an independent external test cohort consists of video images. Because diagnosis with still B-mode images should be quite difficult for humans, even for experts, we applied video images for human diagnoses, whereas the AI diagnosis was based on still images with the “ ≥ 3 out of the 5-frame” rule. Interestingly, the median overall accuracies (percentages of correct answers) were 67.3% and 47.3% for human experts and non-experts, respectively, whereas these were 80.0% for AI model-1, 81.8% for AI model-2, and 89.1% for AI model-3. In addition, all AI models exceeded the performance of human physicians for diagnosing malignant tumors in the comparative study.

Although the overall accuracies for the discrimination of four tumor types was high with the AI models, some tumors were still misdiagnosed even with model-3. The images of six tumors misdiagnosed by AI model-3 are shown in Supplementary Fig. 5. Among these tumors, two were metastatic tumors and four were HCCs. As AI automatically learn the specific features of images, a large number of image data should be required for training of neural network, especially for the diagnosis of lesions with a lot of variation, such as metastatic tumors. Indeed, as shown in Supplementary Fig. 3, although increase of the training improve the diagnostic accuracies, sensitivity of 62.8% for metastatic tumor even by model-3 is still unsatisfactory (Table 1). It is possible that enough amount of training with additional data, such as blood chemical test, may improve the diagnosis for metastatic tumors. On the other hand, in the four HCC nodules misdiagnosed with AI model-3, three showed high estimated probability for HCC, where 97.2% (tumor no. 47), 64.4% (no. 52), and 62.6% (no. 55) were indicated, but failed to present a correct answer under the “ > 3 out of the 5-frame” rule because of the fluctuation of estimated probabilities for HCC among five frames. Further improvement should be required to present the best results.

Because the AI diagnosis is based on the “ ≥ 3 out of the 5-frame” rule for each nodule, we compared the median estimated probabilities for correct answers on the nodules and found that the probabilities substantially improved in most nodules in model-2 and model-3 as compared with model-1. When we compared the estimated probability among the types of tumors, considerable improvement was observed in the diagnosis of HCCs and hemangiomas. For cysts, the probability is 100% in all the estimations, suggesting that the image features are homogeneous and completely different from those of other types of tumors. On the other hand, the insufficient estimated probability for metastatic tumors is one of the limitations of this study. Nevertheless, the AI models, particularly model-3, showed a higher performance in the diagnosis of four types of tumors and malignant tumors as compared with physicians, including qualified experts.

To know the association between performance of AI and size of lesions is intriguing, which has not clarified yet. Yang et al., reported that accuracies for diagnosing malignant tumor were similar regardless of tumor size in internal cohort, but slightly lower in large tumor in external cohort [20]. It is possible that larger tumors may have more variation in image than smaller lesions, which can result in the decrease of performance. We have examined 55 tumors for the performance of AI model; six tumors are not correctly diagnosed by model-3 (Supplementally Fig. 5), where no clear trend in size is observed. Further study using large size of external test cohort should be required to know the effect of tumor size on the performance of AI.

In our AI models, other types of rare tumors were not used as training data, which is another limitation. However, considering that HCC, metastatic tumors, hemangiomas, and cysts are the main constituents of liver tumors, and AI diagnosis is an assistive tool for US examination performed by humans, our models can offer sufficient support for the improvement of human diagnosis in B-mode US.

The AI models in this study focused on the diagnosis, not detection, of tumors, which is also a limitation. A real-time detection system for thyroid tumors has been reported for thyroid US [29, 30]. An AI-US that detects and classifies liver tumors during B-mode examination is under development. We are also conducting a prospective large-scale comparative study between the developed AI-US and human experts for the real-time detection and diagnosis of liver tumors.

## Supplementary Information

Below is the link to the electronic supplementary material.

Supplementary file1 (DOCX 910 KB)

Supplementary file2 (XLSX 14 KB)

Supplementary file3 (DOCX 19 KB)

Supplementary file4 (XLSX 36 KB)

Supplementary file5 (XLSX 33 KB)

Supplementary file6 (DOCX 25 KB)

Supplementary file7 (DOCX 27 KB)
