# Supplementary material for: Artificial intelligence (AI) models for the ultrasonographic diagnosis of liver tumors and comparison of diagnostic accuracies between AI and human experts
Source: J Gastroenterol. 2022 Feb 27;57(4):309–21. doi: 10.1007/s00535-022-01849-9 (PMC8938378; doi:10.1007/s00535-022-01849-9)
Supplement: Supplementary file 1 — Supplementary file1 (DOCX 910 KB) [file 535_2022_1849_MOESM1_ESM.docx]

**Supplementary Figures (Journal of Gastroenterology)**

Artificial intelligence (AI) models for the diagnosis of liver tumors and comparison of diagnostic accuracies between AI and human experts

Naoshi Nishida ^1, *^, Makoto Yamakawa ^2^, Tsuyoshi Shiina ^2^, Yoshito Mekada ^3^, Mutsumi Nishida ^4^, Naoya Sakamoto ^5^, Takashi Nishimura ^6^, Hiroko Iijima ^6^, Toshiko Hirai ^7^, Ken Takahashi ^8^, Masaya Sato ^9, 10^, Ryosuke Tateishi ^10^, Masahiro Ogawa ^11^, Hideaki Mori ^12^, Masayuki Kitano ^13^, Hidenori Toyoda ^14^, Chikara Ogawa ^15^, and Masatoshi Kudo ^1, *^, JSUM AI investigators

**Corresponding Author:**

**Naoshi Nishida: Department of Gastroenterology and Hepatology, Kindai University Faculty of Medicine, 377-2 Ohno-higashi, Osaka-sayama, Osaka 589-8511, Japan; naoshi@med.kindai.ac.jp**

**Supplementary Fig. 1**

Flow diagram of the selection of image data for the development of AI models.


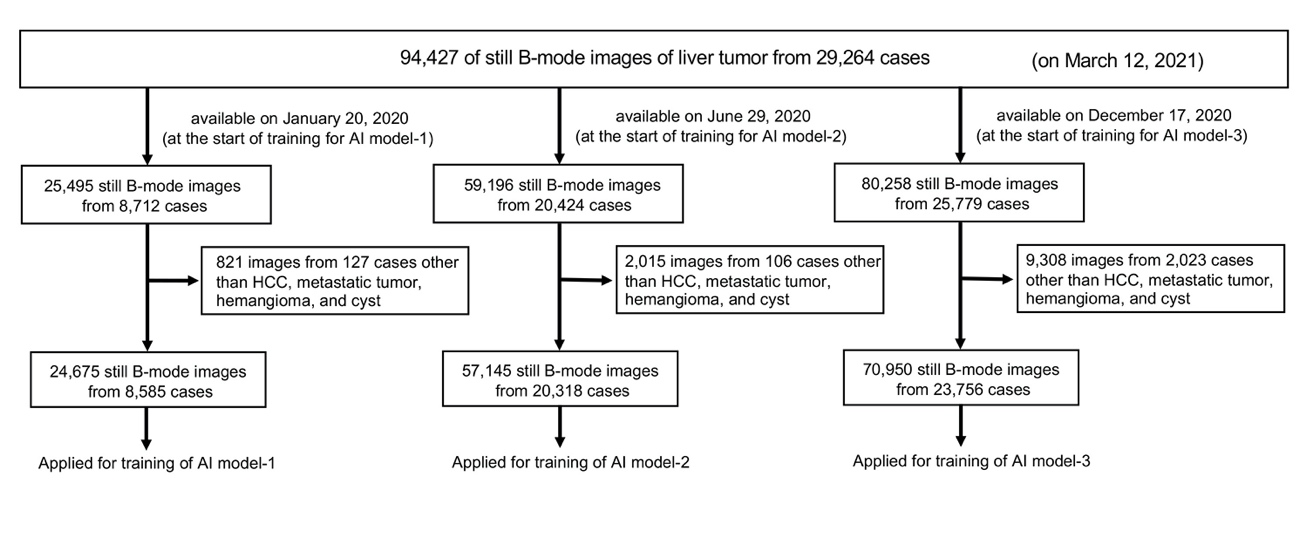


In this study, 94,427 B-mode images of liver tumors from 29,264 cases were collected on March 12, 2021. For AI model-1, 25,495 still B-mode images from 8,712 cases of liver tumors were available by January 20, 2020, of which 24,675 images of four types of liver tumors were applied to develop AI model-1. The remaining 821 images from 127 cases were excluded from the analysis because they presented other types of tumors, which are different from HCC, metastatic tumors, hemangiomas, and liver cysts. Similarly, 59,196 still B-mode images from 20,424 cases were available on June 29, 2020, of which 57,145 images of the four types of liver tumors from 20,318 cases were used for the training of the AI model-2. Then, 80,258 still B-mode images from 25,779 cases were available by December 17, 2020, of which 70,950 images of the four types of liver tumors from 23,756 cases were applied for the training of AI model-3. The remaining 2,015 images from 106 cases collected by June 29, 2020 and 9,308 images from 2,023 cases collected by December 17, 2020 were excluded from the training data for model-2 and model-3, respectively, because they presented other types of tumors.

**Supplementary Fig. 2**

Cropping the ROI, including a liver tumor, from ultrasound images.


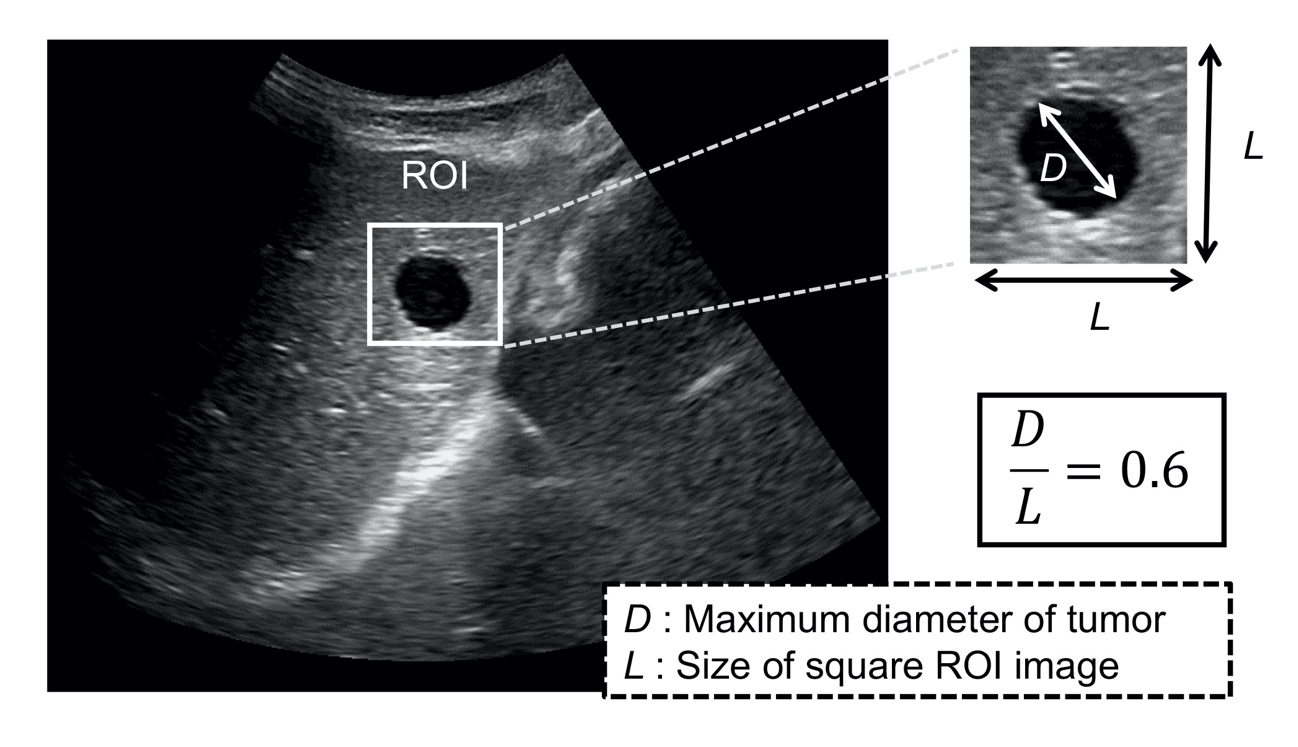


The ROI, including a liver tumor, was cropped from the ultrasound image as a preprocessing step so that the maximum diameter of the tumor (*D*) is 0.6 times the ROI size (*L*).

**Supplementary Fig. 3**

Improve of diagnostic performance of AI models with the increase of the number of training images


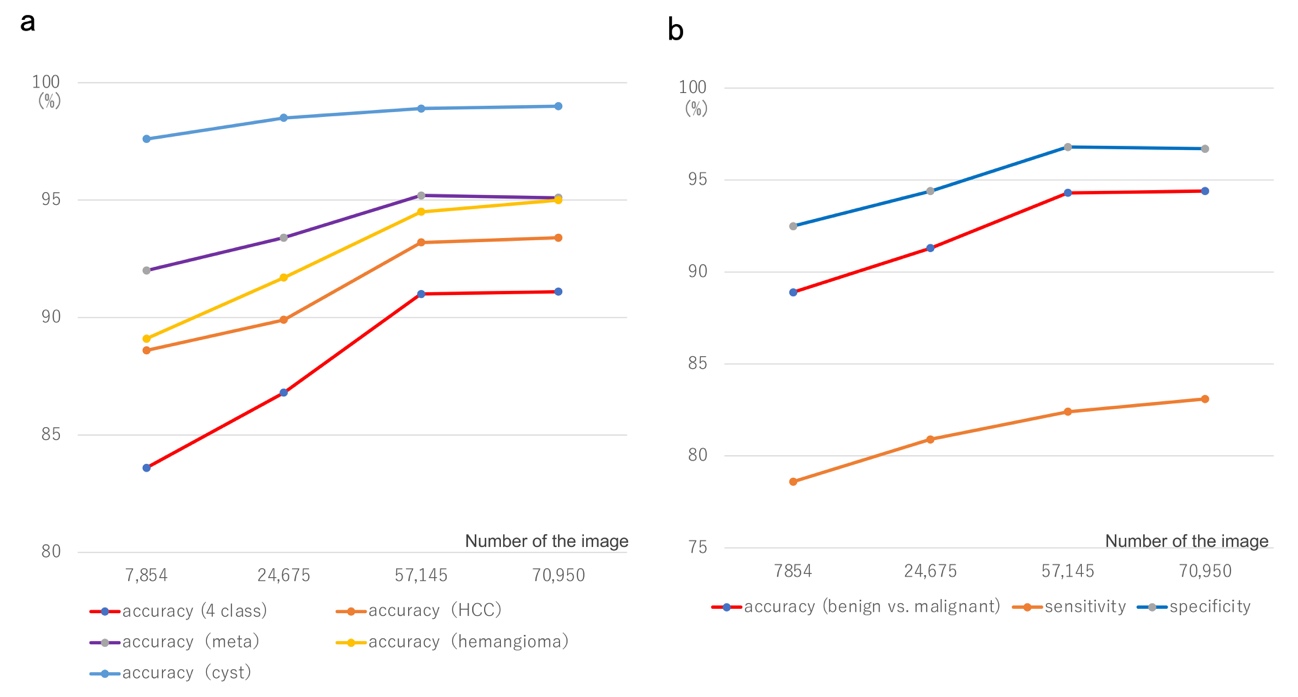


The CNN algorism was trained with 7,854 US images, 24,675 images, 57,145 images, and 70,950 images of liver tumor. The performance of the 4-class discrimination (a) and that of the benign and malignant discrimination (b) are shown. Overall accuracies, and accuracies for diagnosis (percentages of correct diagnoses by AI) of each type of tumors are improved with the increase of training. Similarly, accuracies, sensitivities, and specificities for the diagnosis of malignant tumors are also increased, indicating the training of CNN is effectively performed.

**Supplementary Fig. 4**

US images that show deferent results between AI model-3 and human experts (1)

| tumors | Correct diagnosis | Diagnosis by AI model-3 | US images | Diagnosis by physicians | | | | |
| --- | --- | --- | --- | --- | --- | --- | --- | --- |
|  |  |  |  | expert 1 | expert 2 | expert 3 | expert 4 | expert 5 |
| No. 16 | HCC | hemangioma | 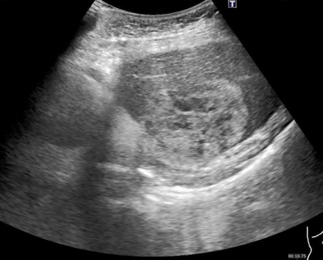 | HCC | meta | hemangioma | HCC | HCC |
| No. 52 | HCC | N.D | 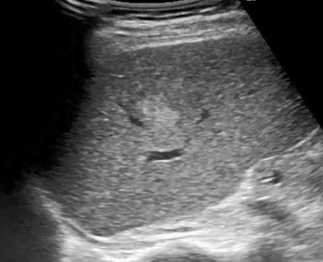 | hemangioma | HCC | HCC | hemangioma | hemangioma |
| No. 55 | HCC | meta | 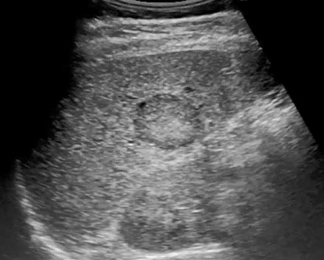 | HCC | HCC | HCC | meta | meta |

Among the 55 cases, six tumors are misdiagnosed by AI model-3. Among them 3 tumors are correctly diagnosed by at least two out of five human experts. The US images of these 3 tumors are shown. Diagnosis shown in red denotes incorrect diagnosis. meta; metastatic liver tumor. N.D; not determined by ">3 out of the five-frame rule" by AI.

US images that show deferent results between AI model-3 and human experts (2)

| Cases | Correct diagnosis | Diagnosis by AI model-3 | US images | Diagnosis by physicians | | | | |
| --- | --- | --- | --- | --- | --- | --- | --- | --- |
|  |  |  |  | expert 1 | expert 2 | expert 3 | expert 4 | expert 5 |
| No. 12 | hemangioma | hemangioma | 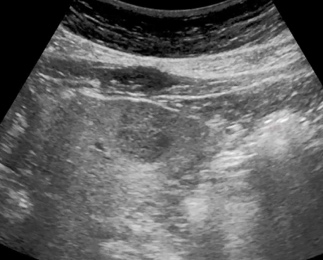 | HCC | HCC | HCC | HCC | HCC |
| No. 15 | hemangioma | hemangioma | 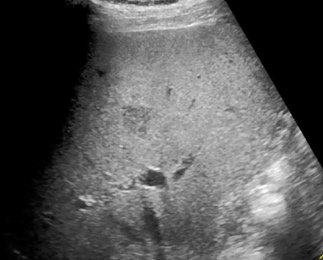 | HCC | HCC | HCC | HCC | HCC |
| No. 24 | meta | meta | 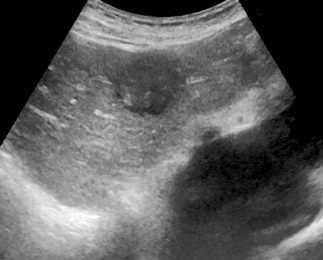 | HCC | HCC | HCC | HCC | HCC |
| No. 30 | HCC | HCC | 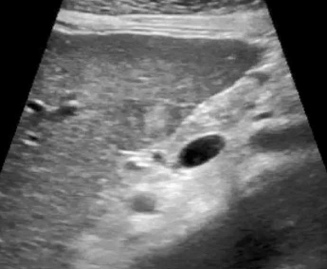 | hemangioma | hemangioma | hemangioma | hemangioma | hemangioma |
| No. 43 | HCC | HCC | 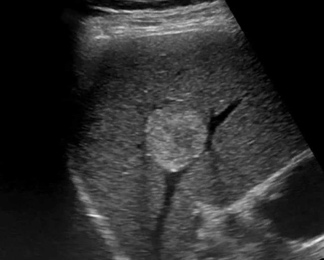 | hemangioma | hemangioma | hemangioma | hemangioma | hemangioma |

Among the 55 cases, five tumors that correctly diagnosed by AI model-3, are misdiagnosed by all five human experts. The US images of these 5 tumors are shown. Diagnosis shown in red denotes incorrect diagnosis. meta; metastatic liver tumor.

**Supplementary Fig. 5**

US images that are misdiagnosed by AI model-3

| Cases | US images | Cases | US images |
| --- | --- | --- | --- |
| No. 16  Correct diagnosis: HCC  Diagnosis by AI model-3  hemangioma | 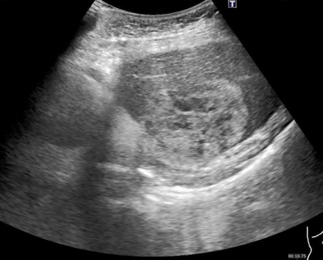 | No. 47  Correct diagnosis: HCC  Diagnosis by AI model-3  N.D | 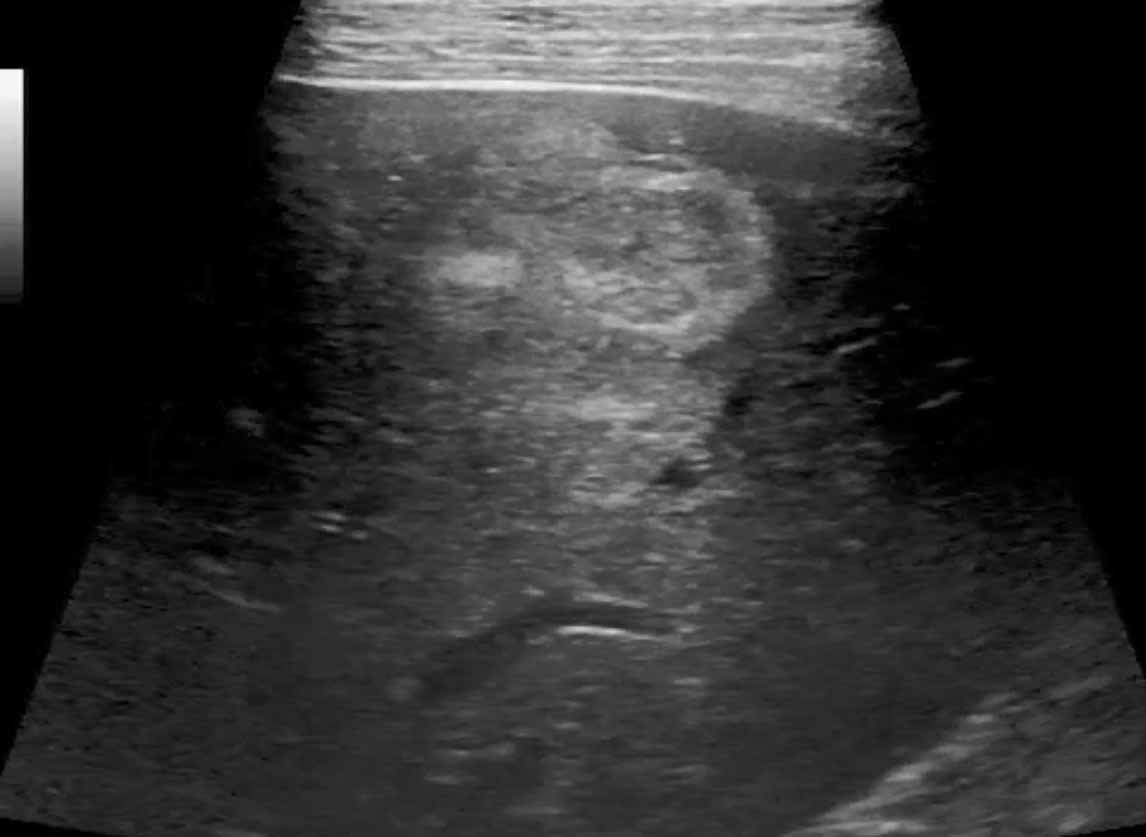 |
| No. 18  Correct diagnosis: meta  Diagnosis by AI model-3  cyst | 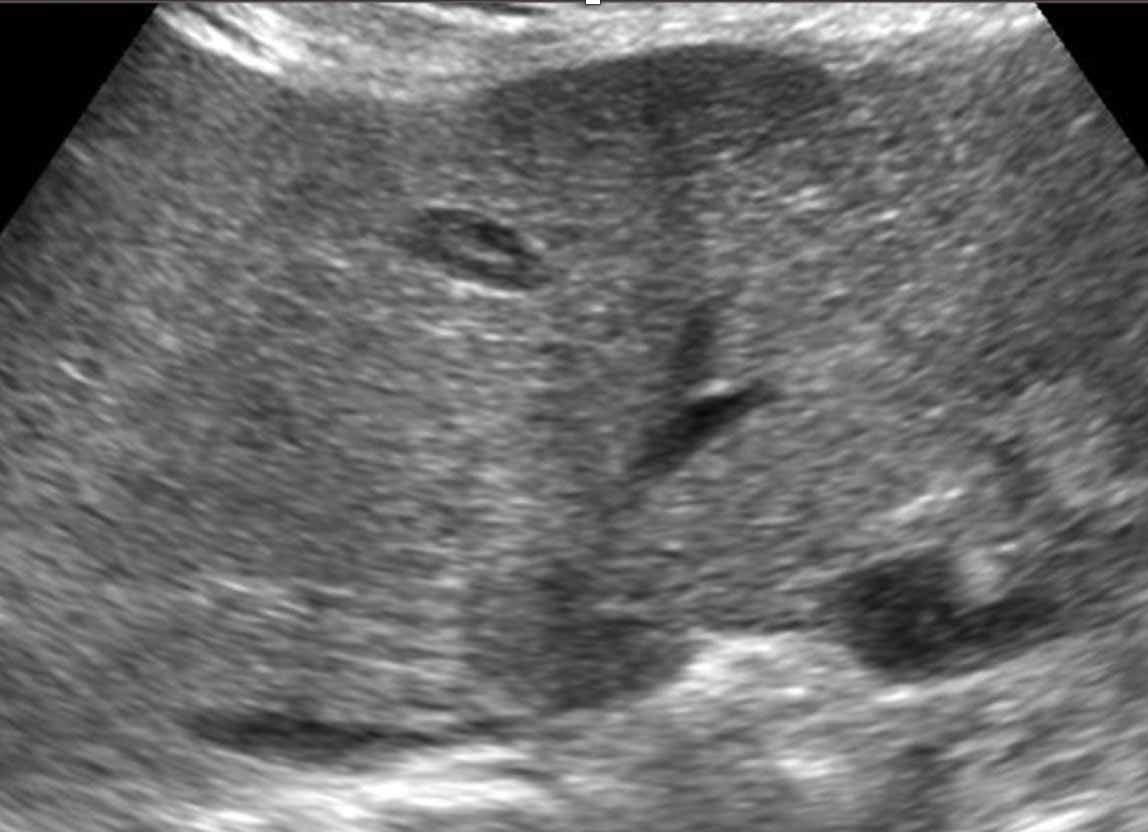 | No. 52  Correct diagnosis: HCC  Diagnosis by AI model-3  N.D | 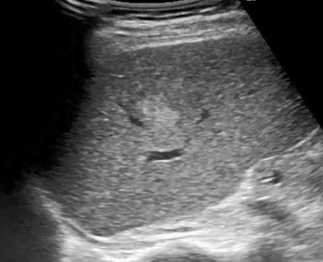 |
| No. 38  Correct diagnosis: meta  Diagnosis by AI model-3  cyst | 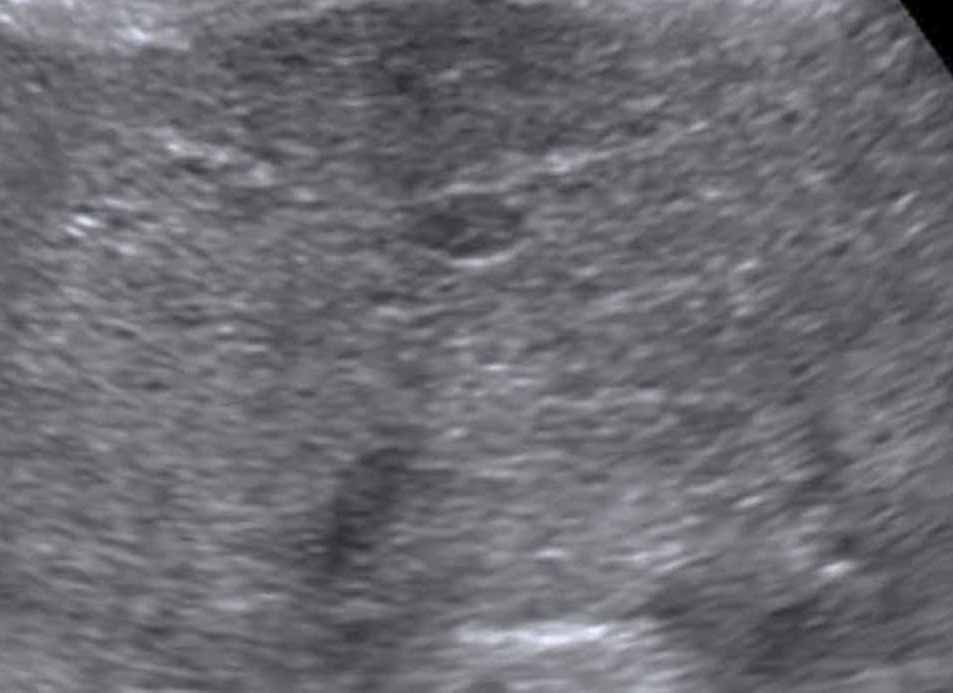 | No. 55  Correct diagnosis: HCC  Diagnosis by AI model-3  meta | 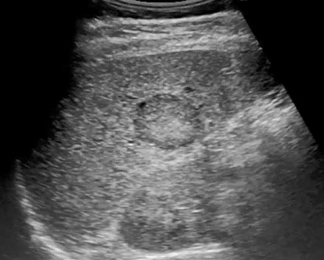 |

Among the 55 lesions, six are misdiagnosed by AI model-3. These six images are shown.

For the tumor no. 16, the correct diagnosis is HCC；a massive high and heterogenous echoic tumor with relatively clear margin is observed. The AI classified this tumor as hemangioma, and the median estimated probability for the diagnosis of HCC by AI is 16.3%. On the other hand, 3 out of 5 human experts diagnosed the tumor as HCC. Other answers by physicians are hemangioma and metastatic tumor.

For the tumor no. 18, the correct diagnosis is metastatic liver tumor; a small hypoechoic tumor with clear margin and target pattern inside is observed. The AI classified this tumor as cyst, and the median estimated probability for the diagnosis of metastatic tumor by AI is 0.4%. No human experts diagnosed this tumor as metastatic tumor. Three experts answered this tumor as a cyst, and two answered as HCC.

For the tumor no. 38, the correct diagnosis is metastatic liver tumor；a small hypoechoic tumor is observed with echogenic pattern (or target pattern) inside the tumor. The AI classified this tumor as cyst, and the median estimated probability for the diagnosis of metastatic tumor by AI is 0.1%. No human experts diagnosed this tumor as metastatic tumor. Three experts answered this tumor as a HCC. Other answers were hemangioma and cyst.

For the tumor no. 47, the correct diagnosis is HCC; the tumor shows clear but irregular margin (possibly multinodular tumor). The inside of the tumor is relatively homogenous with partially strong echo. The median estimated probability for the diagnosis of HCC by AI is 15%. One human expert diagnosed correctly for this tumor. Other 4 experts diagnosed this tumor as hemangioma. Interestingly, among the 5 estimations for this tumor by AI with corresponding 5 frames, 2 estimations indicated this tumor as HCC with 86,1% and 97.2% probability, respectively.

For the tumor no. 52, the correct diagnosis is HCC; a nodular high echoic tumor with irregular and hypoechoic margin is observed. The median estimated probability for the diagnosis of HCC by AI is 4.9%. The range of estimated probability by AI is also large among the frames, where one estimation indicated this tumor as HCC with 64.4% probability. Among the 5 human experts, two correctly answered this tumor as HCC. Other 3 physicians diagnosed this tumor as hemangioma.

For the tumor no. 55, the correct diagnosis is HCC; a nodular high-iso echoic tumor with round shape and hypoechoic margin is observed. The median estimated probability for the diagnosis of HCC by AI is 30.3%. One out of five estimations also indicated this tumor as HCC with 62.6% probability; the range of estimated probability by AI is also large among the frames for this tumor. Among the 5 human experts, three correctly answered this tumor as HCC. Other 2 physicians diagnosed this tumor as metastatic tumor.
