# Supplementary material for: Artificial intelligence (AI) models for the ultrasonographic diagnosis of liver tumors and comparison of diagnostic accuracies between AI and human experts
Source: J Gastroenterol. 2022 Feb 27;57(4):309–21. doi: 10.1007/s00535-022-01849-9 (PMC8938378; doi:10.1007/s00535-022-01849-9)
Supplement: Supplementary file 3 — Supplementary file3 (DOCX 19 KB) [file 535_2022_1849_MOESM3_ESM.docx]

**Supplementary File 2**

**AI model for discriminating liver tumor including intrahepatic cholangiocarcinoma (AI model-ICC)**

1) Training data

| Types of tumors | Number of cases | Number of images |
| --- | --- | --- |
| HCC | 231 | 1,124 |
| ICC | 216 | 1,211 |
| Metastatic tumor | 205 | 1,214 |
| Hemangioma | 320 | 1,114 |
| Total | 972 | 4,663 |

HCC, hepatocellular carcinoma, ICC, intrahepatic cholangiocarcinoma

To avoid a bias based on the disproportion in the member of training images for CNN among tumor types, we downsized the number of the images for HCC, metastatic tumor, and hemangioma to the similar amount of ICC images for training.

2) Performance of AI model-ICC

4-class discrimination

Overall accuracy = 71.5 % (695 / 972)

|  |  | True | | | |
| --- | --- | --- | --- | --- | --- |
|  |  | HCC | ICC | metastatic tumor | hemangioma |
| Estimated | HCC | 136 | 23 | 21 | 23 |
|  | ICC | 18 | 152 | 19 | 10 |
|  | metastatic tumor | 50 | 30 | 141 | 21 |
|  | hemangioma | 27 | 11 | 24 | 266 |
| Accuracy | | 83.3 % | 88.6 % | 83.0 % | 88.1 % |
| Sensitivity | | 58.9 % | 70.4 % | 68.8 % | 83.1 % |
| Specificity | | 91.0 % | 93.8 % | 86.8 % | 90.5 % |

Benign and malignant discrimination (diagnosing malignant tumor)

accuracy = 88.1 % (856 / 972)

sensitivity = 90.5 % (590 / 652)

Specificity = 83.1 % (266 / 320)

|  |  | True | |
| --- | --- | --- | --- |
|  |  | Benign | Malignant |
| Estimated | Benign | 266 | 62 |
|  | Malignant | 54 | 590 |
| Total | | 320 | 652 |
