# Supplementary material for: Artificial intelligence (AI) models for the ultrasonographic diagnosis of liver tumors and comparison of diagnostic accuracies between AI and human experts
Source: J Gastroenterol. 2022 Feb 27;57(4):309–21. doi: 10.1007/s00535-022-01849-9 (PMC8938378; doi:10.1007/s00535-022-01849-9)
Supplement: Supplementary file 6 — Supplementary file6 (DOCX 25 KB) [file 535_2022_1849_MOESM6_ESM.docx]

**Supplementary File 5**

Previous reports for diagnosis of liver tumor based on the machine learning using B-mode images of ultrasonography

| Algorism for machine learning | data sets | Performance | References |
| --- | --- | --- | --- |
| Pre-trained AI using B-mode US images | |  |  |
| ANN | hemangioma: 50  HCC: 87  metastatic tumor: 56 | AUC  0.92 for benign vs. malignant  0.93 for hemangioma vs. HCC  0.94 for hemangioma vs. metastatic tumor | Yoshida H, et al., 2003 ^1^ |
| Two step NN | 65 typical and 46 atypical images from 88 subjects | overall accuracy  90.3% for typical cases  77.5% for atypical cases | Mittal D, et al., 2011 ^2^ |
| SVM | HCC: 27  metastatic tumor: 24  surrounding liver: 54 | overall accuracy: 91.6 %  sensitivity: 90 % for HCC,  93.3 % for metastatic carcinoma | Virmani J, et al., 2013 ^3^ |
| PCA and BPNN | normal liver: 15  cirrhotic liver: 16  HCC: 25 | overall accuracy: 87.7 %  sensitivity: 82.5% for normal liver,  96% for cyst,  93.3% for hemangioma,  90% for HCC,  82.2% for metastatic tumor | Virmani J, et al., 2013 ^4^ |
| PCA and NN | normal liver: 21  cyst: 12  hemangioma: 15  HCC: 28  metastatic carcinoma: 35 | overall accuracy: 95 % | Virmani J, et al., 2014 ^5^ |
| PCA and ANN | cyst; 29  hemangioma: 37  malignant tumor: 33 | overall accuracy: 96%  cyst vs. hemangioma  accuracy: 99.7%  cyst vs. malignant  accuracy: 98.7%  hemangioma vs. malignant  accuracy: 96.1% | Hwang YN et al., 2015 ^6^ |
| ANN (sparse autoencoder) | normal liver: 16  cyst: 44  hemangioma: 18  HCC: 30 | overall accuracy: 97.2%  sensitivity: 91.6%  specificity: 88.5% | Tarek M, et al., 2017 ^7^ |
| CNN | 367 US images  nontumorus liver: 258  hemangioma: 17  metastatic tumor: 48  HCC: 6  cyst: 30  focal nodular hyperplasia: 8 | AUC for tumor detection: 0.935  AUC for tumor discrimination (average of 5 kinds of lesions): 0.916 | Schmauch B, et al., 2019 ^8^ |
| CNN | 24,343 US images of non-cystic FLL (malignant: 2016 patients, benign: 427 patients) | Classification of malignant from benign  accuracy (int. validation): 0.860-0.875,  accuracy (ext. validation): 0.835-0.941  sensitivity (int. validation): 0.855-0.906,  sensitivity (ext. validation): 0.835-0.941  specificity (int. validation): 0.795-1.000,  specificity (ext. validation): 0.500-0.939 | Yang Q, et al., 2020 ^9^ |
| CNN | HCC: 48 | recognition of HCC with B-mode US  AUC: 0.839-0.904  accuracy: 0.829-0.905  sensitivity: 0.677-0.952  specificity: 0.807-0.943 | Mitrea D, et al., 2021 ^10^ |
| Transfer learning | Transfer learning from CE-US images | accuracy: 0.882 ± 0.316,  sensitivity 0.870 ± 0.477,  specificity 0.894 ± 0.377 | Zhang H, et al., 2021 ^11^ |

Reference

1. Yoshida H, et al., Wavelet-packet-based texture analysis for differentiation between benign and malignant liver tumors in ultrasound images. Phys Med Biol. 2003 Nov 21;48(22):3735-53. doi: 10.1088/0031-9155/48/22/008.
2. Mittal D, et al., Neural network based focal liver lesion diagnosis using ultrasound images. Comput Med Imaging Graph. 2011 Jun;35(4):315-23. doi: 10.1016/j.compmedimag.2011.01.007.
3. Virmani J, et al., Characterization of primary and secondary malignant liver lesions from B-mode ultrasound. J Digit Imaging. 2013 Dec;26(6):1058-70. doi: 10.1007/s10278-013-9578-7.
4. Virmani J, et al., A comparative study of computer-aided classification systems for focal hepatic lesions from B-mode ultrasound. J Med Eng Technol. 2013 May;37(4):292-306. doi: 10.3109/03091902.2013.794869.
5. Virmani J, et al., Neural network ensemble based CAD system for focal liver lesions from B-mode ultrasound. J Digit Imaging. 2014 Aug;27(4):520-37. doi: 10.1007/s10278-014-9685-0.
6. Hwang YN, et al., Classification of focal liver lesions on ultrasound images by extracting hybrid textural features and using an artificial neural network. Biomed Mater Eng. 2015;26 Suppl 1:S1599-611. doi: 10.3233/BME-151459.
7. Tarek M, et al., Diagnosis of Focal Liver Diseases Based on Deep Learning Technique for Ultrasound Images. Arabian Journal for Science and Engineering volume, 2017; 42: 3127–3140.
8. Schmauch B, et al., Diagnosis of focal liver lesions form ultrasound using deep learning. Diagn Interv Imaging, 2019 Apr;100(4):227-233. doi: 10.1016/j.diii.2019.02.009.
9. Yang Q, et al., Improving B-mode ultrasound diagnostic performance for focal liver lesions using deep learning: A multicentre study. EBioMedicine. 2020 Jun;56:102777. doi: 10.1016/j.ebiom.2020.102777.
10. Mitrea, D et al., Hepatocellular Carcinoma Automatic Diagnosis within CEUS and B-Mode Ultrasound Images Using Advanced Machine Learning Methods. Sensors (Basel). 2021 Mar 21;21(6):2202. doi: 10.3390/s21062202.
11. Zhang H, et al., Multi-Source Transfer Learning Via Multi-Kernel Support Vector Machine Plus for B-Mode Ultrasound-Based Computer-Aided Diagnosis of Liver Cancers. IEEE J Biomed Health Inform. 2021 Oct;25(10):3874-3885. doi: 10.1109/JBHI.2021.3073812.
