# Supplementary material for: Artificial intelligence (AI) models for the ultrasonographic diagnosis of liver tumors and comparison of diagnostic accuracies between AI and human experts
Source: J Gastroenterol. 2022 Feb 27;57(4):309–21. doi: 10.1007/s00535-022-01849-9 (PMC8938378; doi:10.1007/s00535-022-01849-9)
Supplement: Supplementary file 7 — Supplementary file7 (DOCX 27 KB) [file 535_2022_1849_MOESM7_ESM.docx]

*Supplementary materials and method*s *(Journal of Gastroenterology)*

Artificial intelligence (AI) models for the diagnosis of liver tumors and comparison of diagnostic accuracies between AI and human experts

Naoshi Nishida ^1, *^, Makoto Yamakawa ^2^, Tsuyoshi Shiina ^2^, Yoshito Mekada ^3^, Mutsumi Nishida ^4^, Naoya Sakamoto ^5^, Takashi Nishimura ^6^, Hiroko Iijima ^6^, Toshiko Hirai ^7^, Ken Takahashi ^8^, Masaya Sato ^9, 10^, Ryosuke Tateishi ^10^, Masahiro Ogawa ^11^, Hideaki Mori ^12^, Masayuki Kitano ^13^, Hidenori Toyoda ^14^, Chikara Ogawa ^15^, and Masatoshi Kudo ^1, *^, JSUM AI investigators

**Corresponding Author:**

**Naoshi Nishida: Department of Gastroenterology and Hepatology, Kindai University Faculty of Medicine, 377-2 Ohno-higashi, Osaka-sayama, Osaka 589-8511, Japan; naoshi@med.kindai.ac.jp**

**Construction of AI model using convolutional neural network**

Dataset for construction of AI models

In this study, 94,427 B-mode images of liver tumors from 29,264 cases were collected from April 1, 2018 to March 12, 2021 in 11 core medical centers across Japan: Kindai University Hospital, Hokkaido University Hospital, Hyogo College of Medicine College Hospital, Nara Medical University Hospital, Kyoto University Hospital, the University of Tokyo Hospital, Nippon University Hospital, Kyorin University Hospital, Wakayama Medical University Hospital, Ogaki Municipal Hospital, and Takamatsu Red Cross Hospital.

Using these images, three CNN AI models were constructed for descrimination of 4 types of liver tumor (HCC, metastatic tumor, hemangioma, and cyst): 24,675 still US images of liver tumors from 8,585 cases (4,849 images of HCC from 1,289 cases, 3,668 images of metastatic tumors from 693 cases, 8,279 images of hemangioma from 2,818 cases, and 7,879 images of cysts from 3,785 cases) were used for training, validation and test cohorts, with a ratio of 8:1:1 for training, validation and test cohorts, respectively) for model-1. For model-2, 57,145 images of liver tumors from 20,318 cases (8,557 images of HCC from 2,249 cases, 7,016 images of metastatic tumors from 1,265 cases, 18,464 images of hemangioma from 5,473 cases, and 23,108 images of cysts from 11,311 cases) were used. For model-3, 70,950 images of liver tumors from 23,756 cases (10,388 images of HCC from 2,593 cases, 8,712 images of metastatic tumors from 1,493 cases, 23,829 images of hemangioma from 6,710 cases, and 28,011 images of cysts from 12,960 cases) were used.

We also constracted the AI model for descrimination of another set of of liver tumors (HCC, intrahepatic cholangiocarcinoma [ICC], metastatic tumor, and hemangioma), although the data is still preliminary because of the lack of the number of cases with ICC. For the training of the US image of ICC, 1,211 images from 216 cases are applied. In order to avoid the biased diagnosis based on the disproportion among that number of training images from each type of tumor, we downsized the training data of HCC, metastatic tumor, and hemangioma to 1,124 image from 231 cases, 1,214 imaes from 205 cases, and 1,114 images form 320 cases, respectively.

We uploaded the research information on the web at https://www.med.kindai.ac.jp/shoukaki/research/AI%20data%20base.pdf on Sep. 19, 2018 (revised on Apr. 13, 2020) to inform the patients of the study purpose, eligibility criteria, data collection, hospitals involved in the study, contact information for inquiry, and exclusion criteria for data collection.

**Comparative study of discrimination of liver tumors between AI and phisicians**

*Dataset for comparative study*

A comparative study was conducted using video images from 55 cases of liver tumors. The test dataset used for comparison of AI and human experts was totally different from those used for the cross-validation above. The tumor diagnoses were confirmed by histology, CECT, or Gd-EOB-DTPA-enhanced MRI. The number of each tumor type used in this study is as follows: 18 nodules of HCC, 9 nodules of metastatic tumor, 23 nodules of hemangioma, and 5 nodules of cyst. All tumors used for this study are the nodular type with visible boundary. After US examination of a liver tumor, each video was saved in MP4 video format. Video images showing an entire picture of a tumor were selected for the comparison test between AI models and human. The median size of the tumors is 22 mm in the major axis (range: 6–75). The median size of each type of tumor is as follows: 26 mm (11–63) for HCC, 22 mm (9–75) for metastatic tumor, 18 mm (8–59) for hemangioma, and 23 mm (6–46) for cyst. The median frame rate (fps: frame per second) of the examination is 20 fps (13–47). The length of the video image ranges from 4 to 7 seconds. The US examinations and acquisition of images were performed using LOGIQ S8 and LOGIQ E9 (GE Healthcare, Chicago, IL), and Aplio i800 (Canon Medical Systems Co., Ohtawara, Japan).

*AI models used for comparison between AI and physicians*

As mentioned above, the 10-fold cross-validation method was used, indicating that 10 pretrained CNN models were created for model-1, model-2, and model-3, respectively. If one of the 10 models created by the cross-validation is used for comparison between AI and humans, only 80% of the data will be used for training. To make effective use of the data, we estimated the probability of diagnosis in the comparison study using three CNN models. This is because all the data used for cross-validation can be used as training data in any of the three models. Then, the average probabilities of the three CNN models were used as the estimated probabilities of the AI models and for comparison of diagnostic accuracy of AI and humans. The liver tumor type with the highest average probability was considered to be the estimated result of the AI model.

*Diagnosis of tumor by AI and physicians*

The tumor diagnosis was performed by physicians from four types of tumors using video images. The sites of the tumor in the images were indicated in the video. Eight physicians participated in this study: five were experts who were qualified as specialists by the Japan Society of Gastroenterology (JSGE), the Japan Society of Hepatology (JSH), and/or the Japan Society of Ultrasonic Medicine (JSUM) (expert group), whereas the remaining three were non-specialists (non-expert group). There was no time restriction for answers for the physicians. The status of qualifications and years after graduation of each physician are listed below.

| Human physicians | Years after graduation | Qualification | | |
| --- | --- | --- | --- | --- |
|  |  | JSGE | JSH | JSUM |
| Expert 1 | > 10 years | yes | yes | yes |
| Expert 2 | > 10 years | yes | yes | yes |
| Expert 3 | > 10 years | yes | yes | yes |
| Expert 4 | > 10 years | yes | yes | yes |
| Expert 5 | 5 - 10 years | yes | yes | No |
|  |  |  |  |  |
| Non- expert 1 | 5 - 10 years | No | No | No |
| Non- expert 2 | 3 - 5 years | No | No | No |
| Non- expert 3 | < 2 years | No | No | No |

On the other hand, for the diagnosis using AI, still images of the video frames were used. In total, five different frames showing tumors were selected for each nodule; one is a frame showing the tumor with the maximum diameter, two showing tumors of 75 % of the maximum diameter, and two with tumors of 50 % of the maximum diameter. The site of the tumor was selected as a ROI in the frame and used for the AI diagnosis as described previously. Among the four types of tumors, the diagnosis with the highest estimated probability in three or more frames was selected as the AI diagnosis (> 3 out of the 5-frame rule).
